# Antiviral efficacy of heparan sulfate and enoxaparin sodium against SARS‐CoV‐2

**DOI:** 10.1002/ardp.202400545

**Published:** 2024-11-09

**Authors:** Virginia Fuochi, Salvatore Furnari, Giuseppe Floresta, Vincenzo Patamia, Chiara Zagni, Filippo Drago, Antonio Rescifina, Pio Maria Furneri

**Affiliations:** ^1^ Department of Biomedical and Biotechnological Sciences (Biometec) University of Catania Catania Italy; ^2^ Department of Drug and Health Sciences (DSFS) University of Catania Catania Italy

**Keywords:** absorption inhibition, ACE2, glycosaminoglycans, molecular docking, SARS‐CoV‐2

## Abstract

As the world transitions from the acute phase of the COVID‐19 pandemic caused by SARS‐CoV‐2, the scientific community continues to explore various therapeutic avenues to control its spread and mitigate its ongoing effects. Among the promising candidates are heparan sulfate (HS) and enoxaparin (EX), which have emerged as potential virus inhibitors. HS, a type of glycosaminoglycan, plays a prominent role in the attachment of the virus to host cells. At the same time, EX, a low‐molecular‐weight heparin, is being investigated for its ability to disrupt the interaction between the spike protein of SARS‐CoV‐2 and the ACE2 receptor in human cells. Understanding the mechanisms through which these substances operate could lay the foundation for new strategies in the ongoing management of COVID‐19. This study aimed to examine the details of SARS‐CoV‐2's entry mechanisms and the role of HS in this process. Furthermore, it examines EX's mechanism of action, highlighting how it potentially inhibits SARS‐CoV‐2. The interactions between HS and the virus, alongside in‐vitro and in‐silico inhibition studies with HS and EX, are critically analyzed to assess their antiviral efficacy. Additionally, the antiviral activity of sulfated polysaccharides and the potential therapeutic applications of these findings are discussed.

## Graphical abstract

Heparan sulfate (HS) and enoxaparin (EX) are potential virus inhibitors for treating COVID‐19. HS aids virus attachment to host cells, while EX disrupts the SARS‐CoV‐2 spike protein‐ACE2 receptor interaction. These mechanisms are investigated by analyzing in‐vitro and in‐silico data on their antiviral efficacy and discussing the therapeutic potential of sulfated polysaccharides.

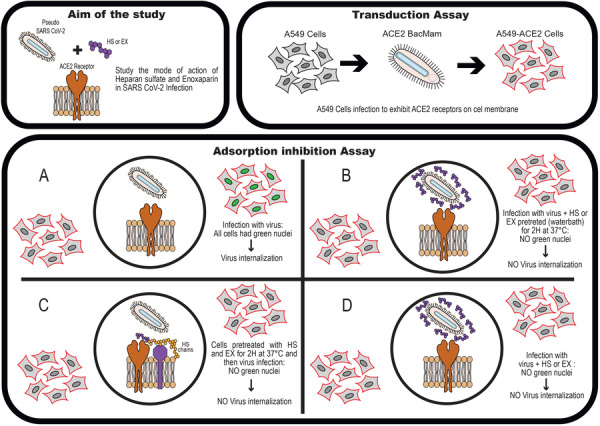

## 1 INTRODUCTION

Beta coronaviruses represent a significant group of viruses that can cause severe respiratory diseases in humans. They have been responsible for notable outbreaks, including severe acute respiratory syndrome (SARS), Middle East respiratory syndrome (MERS), and the ongoing coronavirus disease 2019 (COVID‐19) pandemic.^[^ ^1^ ^]^

The interaction between beta coronaviruses and specific cellular receptors is important for understanding their pathogenesis and devising potential therapeutic interventions. Each coronavirus strain demonstrates a unique preference for cellular receptors, contributing to differences in infectivity and disease severity.^[^ ^2^ ^]^ For instance, while some beta coronaviruses like HCoV‐OC43 and HCoV‐HKU1 interact with 9‐O‐Ac‐Sia,^[^ ^3^, ^4^ ^]^ others such as SARS‐CoV‐1, SARS‐CoV‐2, and MERS‐CoV prefer the ACE2 receptor.^[^ ^5^ ^]^ This selectivity in receptor binding is mediated by the spike (S) glycoproteins on the viral envelope. Specifically, the receptor‐binding domain (RBD) of the spike protein in SARS‐CoV‐1 and SARS‐CoV‐2 recognizes ACE2, allowing the virus to enter host cells and initiate infection.^[^ ^6^ ^]^ Understanding these interactions at the molecular level is vital for developing targeted therapies and vaccines. Interventions that disrupt the binding between the virus and its cellular receptor could inhibit viral entry and reduce the severity of infection. Additionally, knowledge of the cellular receptors targeted by different coronaviruses can inform the development of broad‐spectrum antiviral drugs that may be effective against multiple strains.

An emerging strategy in combating SARS‐CoV‐2 involves investigating the interaction between the virus and glycosaminoglycans (GAGs), which are key for viral attachment, entry, and infection.^[^ ^3^, ^7^, ^8^ ^]^ GAGs include dermatan sulfate, keratan sulfate, heparin (HP), and heparan sulfate (HS).^[^ ^9^ ^]^ Enoxaparin (EX), a molecular fragment extracted from heparin, and HS are the most abundant GAGs in the human body, constituting 50%–90%.^[^ ^10^ ^]^ Both are attached to the surface of endothelial cells as part of the glycocalyx, a structure encompassing free glycans, glycoproteins, proteoglycans, and glycolipids.^[^ ^11^ ^]^

HS has been identified as necessary in the initial stages of viral infection, particularly in SARS‐CoV‐2.^[^ ^12^ ^]^

Studies have shown that GAGs, including HS and EX, are necessary for the infectivity of various viruses such as Herpes simplex virus,^[^ ^13^ ^]^ Dengue virus,^[^ ^14^ ^]^ Echovirus,^[^ ^15^ ^]^ and North American eastern equine encephalitis virus.^[^ ^16^ ^]^ Laboratory experiments have also highlighted a correlation between GAGs and viruses like Cytomegalovirus,^[^ ^17^ ^]^ Pseudorabies virus,^[^ ^18^ ^]^ Merkel cell polyomavirus,^[^ ^19^ ^]^ Hepatitis B and Hepatitis Delta virus.^[^ ^20^ ^]^

Understanding the role of GAGs in viral infection is particularly relevant for emerging viral variants like the Omicron variant of SARS‐CoV‐2 and its sublineages, such as BA.2.86. These variants challenge existing therapeutic strategies, emphasizing the need for innovative approaches.

To address this, we employed computational simulations and biological assays to investigate the antiviral activity of GAGs against different strains of SARS‐CoV‐2, including the BA.2.86 variant. This integrated approach allows for a comprehensive evaluation of GAGs' potential as antiviral agents.

Using BacMam technology in *in*‐*vitro* assessments ensures a thorough evaluation of GAGs' effectiveness against SARS‐CoV‐2. This technology enables the transient expression of target proteins in mammalian cells, allowing for the study of viral entry and replication in a controlled environment. By leveraging computational modeling and experimental validation, this research aims to provide a significant understanding of the mechanisms underlying GAG‐mediated inhibition of SARS‐CoV‐2 infection. These findings could lead to the development of novel antiviral therapeutics targeting viral‐host interactions, including those between SARS‐CoV‐2 and GAGs.

## 2 RESULTS AND DISCUSSION

### 2.1 Cell viability

**Figure 1:**
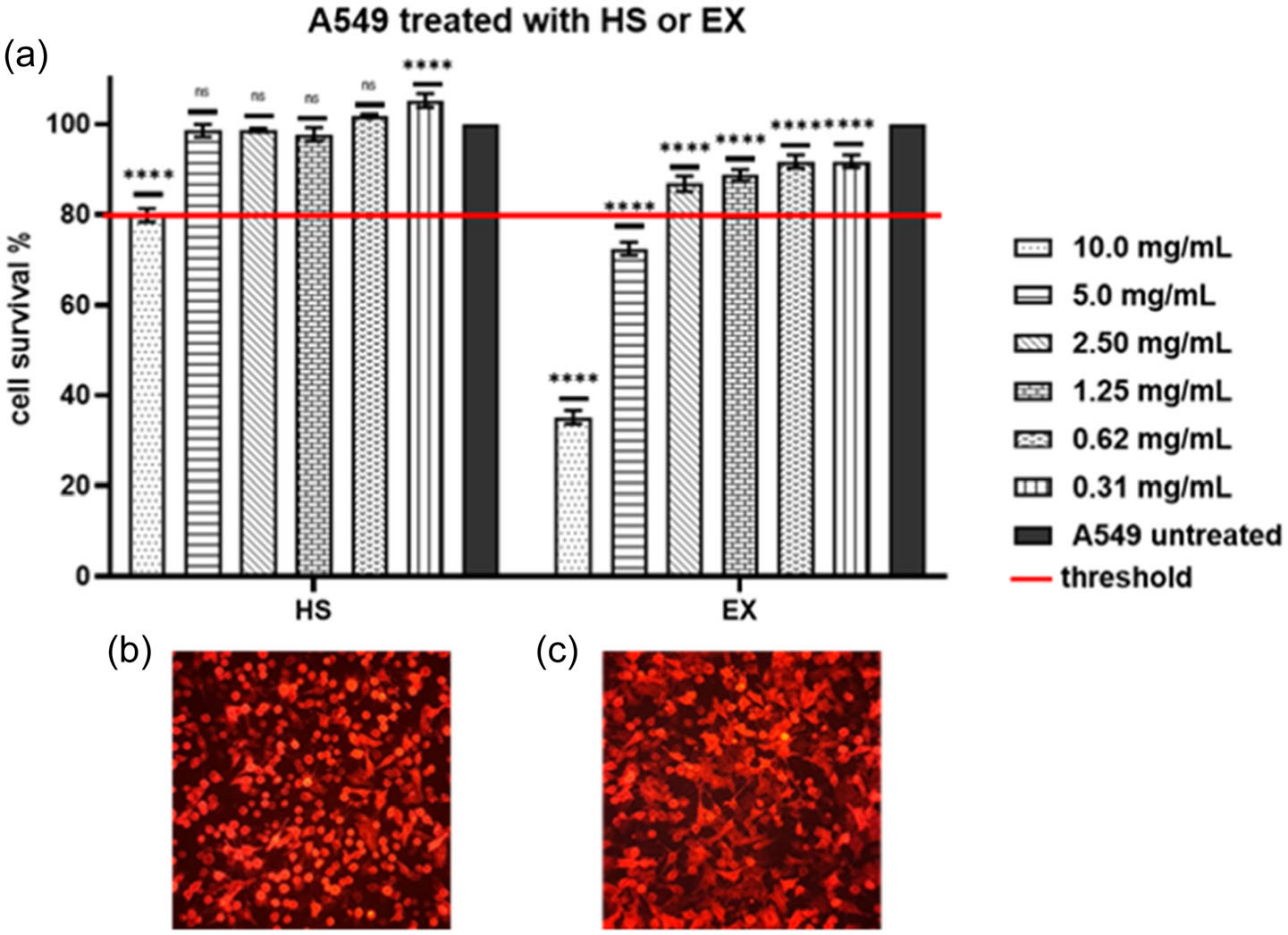
(a) A549 cells were treated with HS (left) and EX (right); (b) A549‐ACE2 cells treated with HS at 5.0 mg/mL that, as shown in the graph above, is the first concentration with a percentage of cells availability over the chosen threshold; (c) A549‐ACE2 cells were treated with EX at 1.25 mg/mL. As shown in the graph above, this is the first concentration with a percentage of cell availability over the chosen threshold. Data were analyzed by two‐way ANOVA with correction for multiple comparisons by Bonferroni (****p < 0.0001).

HS and EX cytotoxicities were evaluated using an MTT assay on the A549 cell line at 24 h. In this assay, different concentrations of HS and EX ranging from 10.0 to 0.31 mg/mL were tested. The results of the MTT assay, as depicted in Figure 1, indicated that HS exhibited minimal cytotoxic effects on the A549 cells. At a 5.0 mg/mL concentration, HS ensured high cell viability well above the chosen reference threshold of 80%, with a cell viability of 98.55%. This suggests that HS is well tolerated by the cells even at relatively high concentrations, indicating its safety profile for potential therapeutic use.

On the other hand, EX showed varying degrees of cytotoxicity depending on the concentration tested. At high concentrations, EX reduced cell viability, indicating cytotoxic effects. However, at a concentration of 2.5 mg/mL, EX was well tolerated by the cells, with a cell viability of 86.84%. This suggests that lower concentrations of EX are safer for the cells, while higher concentrations may induce cytotoxic effects.

Overall, these findings provide valuable information about the safety profiles of HS and EX on the A549 cell line.

These results are essential for determining appropriate dosage levels for further investigation of the antiviral properties of HS and EX and their potential therapeutic applications.

### 2.2 Pseudovirus SARS‐CoV‐2 affinity for ACE2 validation

**Figure 2:**
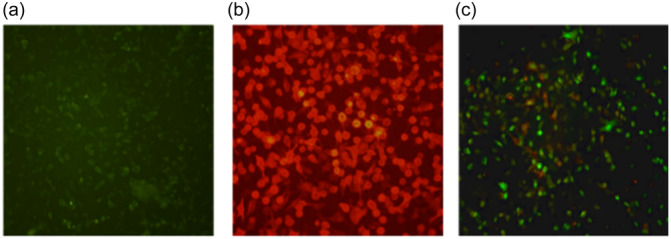
(a) A549 cells not exhibiting ACE2 receptors; (b) A549 cells expressing ACE2 receptors (A549‐ACE2); (c) A549‐ACE2 cells infected with pseudo SARS‐CoV‐2. The infection can be visualized by green staining of cell nuclei.

Before evaluating the effectiveness of HS and EX against the pseudovirus, we confirmed the reliability of our assay by comparing the transduction efficiency of a pseudovirus exposing the SARS‐CoV‐2 spike protein in A549 cells, both with (Figure 2b) and without (Figure 2a) ACE2 receptor expression. As illustrated in Figure 2, the pseudovirus transduction efficiency was higher in A549‐ACE2 cells stably expressing the ACE2 receptor (Figure 2c) compared with normal A549 cells. These findings demonstrate that the entry efficiency of the pseudo‐SARS‐CoV‐2 reporter in A549 cells lacking endogenous ACE2 receptor expression is very low. This validation supports using this system to test the efficacy of HS and EX molecules.

### 2.3 Antiviral activity of HS and EX

As mentioned previously, to determine whether HS and EX block SARS‐CoV‐2 viral entry, we utilized a safe *facsimile* of the virus that does not replicate in human cells and can be used in pseudo‐host cells expressing ACE2. The pseudo‐SARS‐CoV‐2 reporter is a baculovirus decorated with the spike protein, similar to the actual SARS‐CoV‐2 virus, allowing it to be used in our biosafety Level 2 facility. HS and EX were tested using a SARS‐CoV‐2 pseudovirus entry assay, with A549‐ACE2 cells divided into three groups.

**Figure 3:**
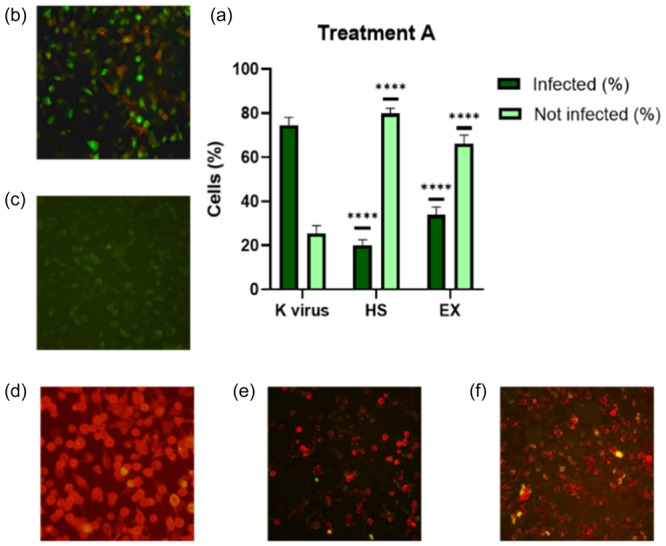
(a) A549‐ACE2 treated with pseudovirus and HS or EX simultaneously; (b) A549‐ACE2 cells infected with pseudovirus; (c) A549 cells not infected; (d) A549 cells transduced with ACE2‐reporter; (e) A549‐ACE2 cells treated with pseudovirus and HS; (f) A549‐ACE2 cells treated with pseudovirus and EX. Data were analyzed by two‐way ANOVA with correction for multiple comparisons by Bonferroni (****p < 0.0001).

In the first group (Treatment A), A549‐ACE2 cells were simultaneously infected with the SARS‐CoV‐2 pseudovirus and treated with either HS (5.0 mg/mL) or EX (1.25 mg/mL). As shown in Figure 3, both substances inhibited viral absorption. Specifically, in Figure 3e‐f, very few infected cells were observed with the co‐treatment of HS and EX. These reductions were statistically significant compared with the virus control group, showing a decrease of about 75% and 50%, respectively.

**Figure 4:**
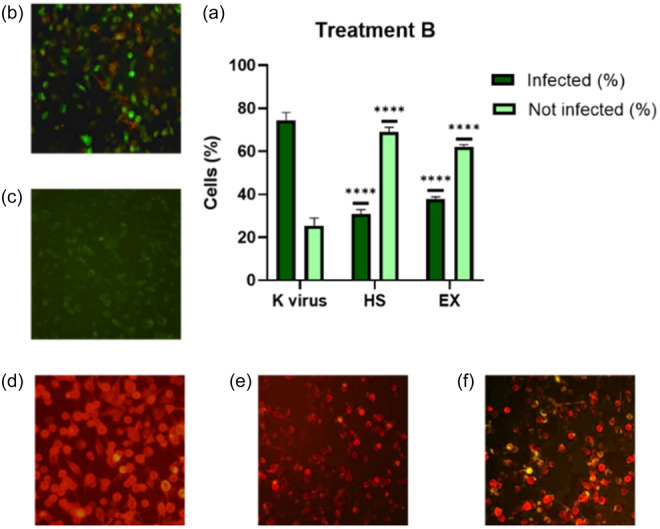
(a) A549‐ACE2 cells pretreated with HS or EX for 2 h at 37°C and then infected with pseudovirus; (b) A549‐ACE2 cells infected with pseudovirus; (c) A549 cells not infected; (d) A549 cells transduced with ACE2‐reporter; (e) A549‐ACE2 cells pretreated with HS; (f) A549‐ACE2 cells pretreated with EX. Data were analyzed by two‐way ANOVA with correction for multiple comparisons by Bonferroni (****p < 0.0001).

The second group (Treatment B) of A549‐ACE2 cells were exposed to HS or EX for 2 h before being infected with SARS‐CoV‐2 reporter. Figure 4 shows that pretreatment with these drugs significantly inhibited viral entry. As in co‐treatment, the antiviral activity in this setup was notably intense: HS inhibited viral absorption of 60% (Figure 4e), while EX reduced viral entry by 48% (Figure 4f).

**Figure 5:**
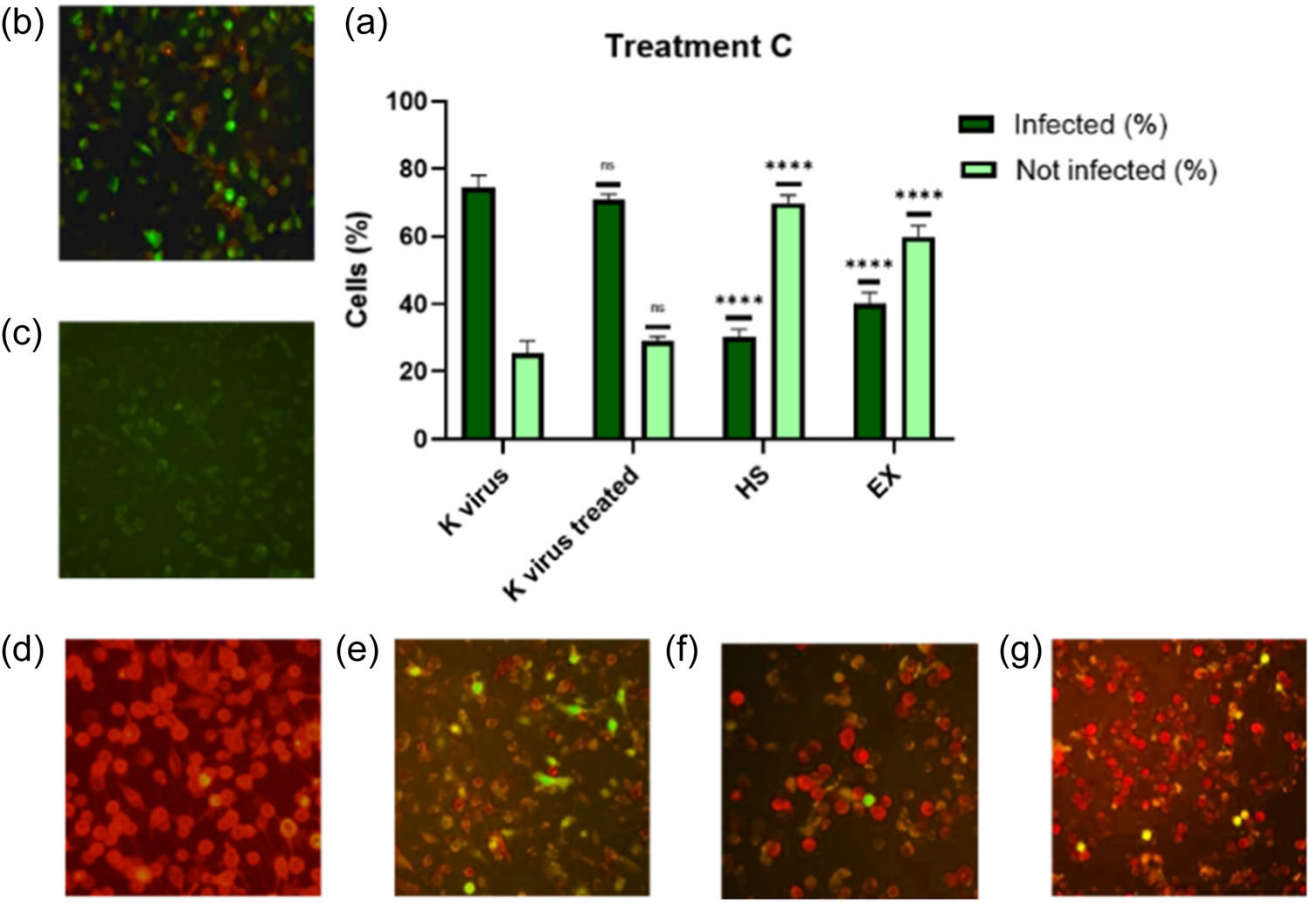
(a) Virus was preincubated with HS or EX for 2 h before being added to A549‐ACE2 cells; (b) A549‐ACE2 cells infected with pseudovirus; (c) A549 cells not infected; (d) A549 cells transduced with ACE2‐reporter; (e) A549‐ACE2 cells infected with pseudovirus pretreated for 2 h at 37°C (water bath) to show the survival of the virus in these conditions; (f) A549‐ACE2 cells infected with pseudovirus pretreated with HS for 2 h at 37°C. Data were analyzed by two‐way ANOVA with correction for multiple comparisons by Bonferroni (****p < 0.0001).

In the last group (Treatment C), the virus was preincubated with HS or EX for 2 h before being added to A549‐ACE2 cells. As depicted in Figure 5a, the A549‐ACE2 cells infected with the virus, which had been pretreated at 37°C for 2 h, demonstrated the virus's persistence and survival under these conditions. In contrast, Figure 5f,g showed no viral infection, indicating that pretreating the virus with HS or EX prevented it from binding to the cellular receptor and entering the cells.

These results demonstrate that the GAGs HS and EX exhibited antiviral activity in all treatment scenarios tested.

### 2.4 Molecular modeling

Molecular modeling experiments were conducted to confirm the interactions of HS and EX at the interface between the spike protein of SARS‐CoV‐2. Initially, we studied their interaction with the spike protein of the wild‐type virus, followed by the highly mutated BA.2.86 variant, and additionally with the two variants KP.2 and JN.1 arising from BA.2.86 that are rapidly spreading in multiple regions as of April 2024.^[^ ^21^ ^]^

**Figure 6:**
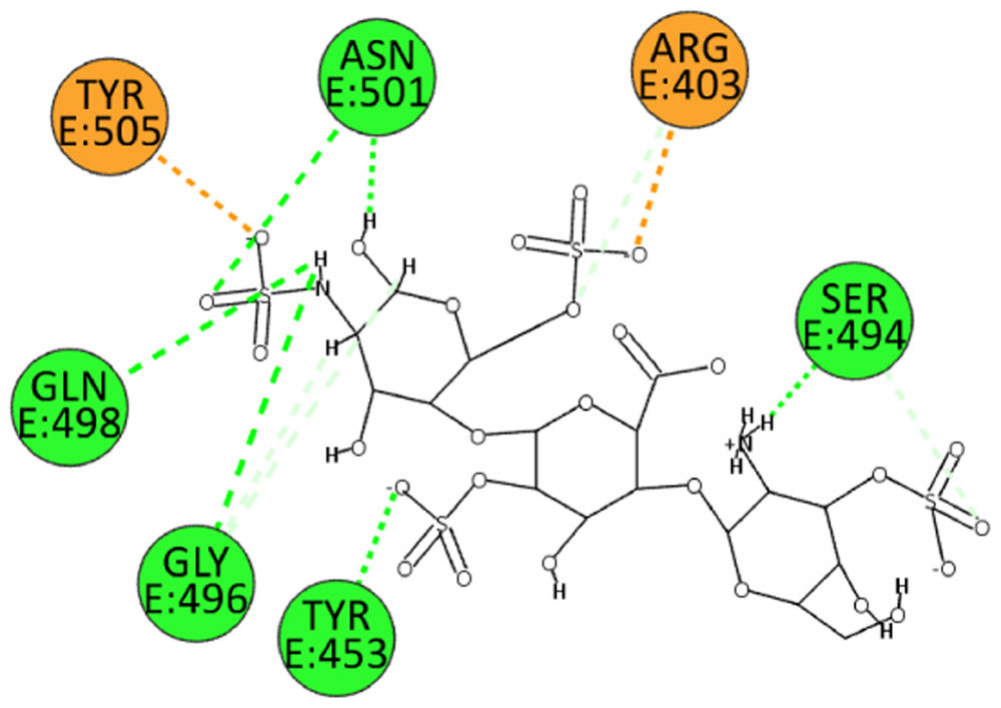
2D pose of HS at the interface between the crystal structures of SARS‐CoV‐2 spike bound to ACE2 (wild type, PDB ID: 6M0J).

Numerous electrostatic interactions were identified for HS with the spike protein of the wild‐type SARS‐CoV‐2. Specifically, HS interacts with residues Arg403 and Tyr505. Additionally, due to several sulfate groups, HS interacts with residues Tyr453, Ser494, Gly496, Gln498, and Asn501 (Figure 6). These interactions suggest a strong binding affinity and a potential mechanism by which HS can inhibit viral entry.

**Figure 7:**
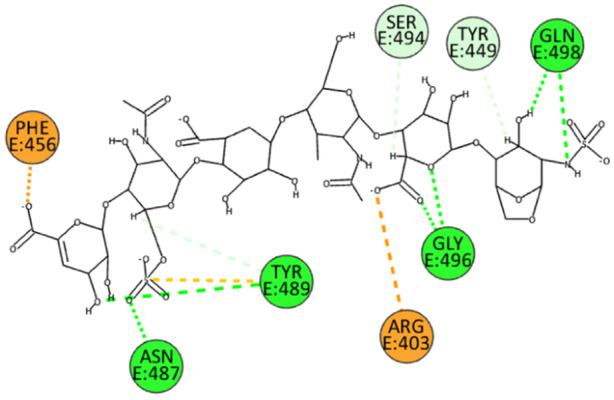
2D pose of EX at the interface between the crystal structures of SARS‐CoV‐2 spike bound to ACE2 (wild type, PDB ID: 6M0J).

For EX, the many sulfate and carboxyl groups influence its binding position. Specifically, the carboxylate ion facilitates the formation of *π*‐anion and electrostatic interactions with residues Arg456 and Arg403. Additionally, the sulfate and hydroxyl groups in EX form several hydrogen bonds, particularly with residues Asn487, Tyr489, Gly496, and Gln498. Unconventional hydrogen bonds are also formed with residues Tyr449 and Ser494 (Figure 7). These interactions highlight the distinct binding characteristics of EX and its potential mechanism for inhibiting viral entry.

**Figure 8:**
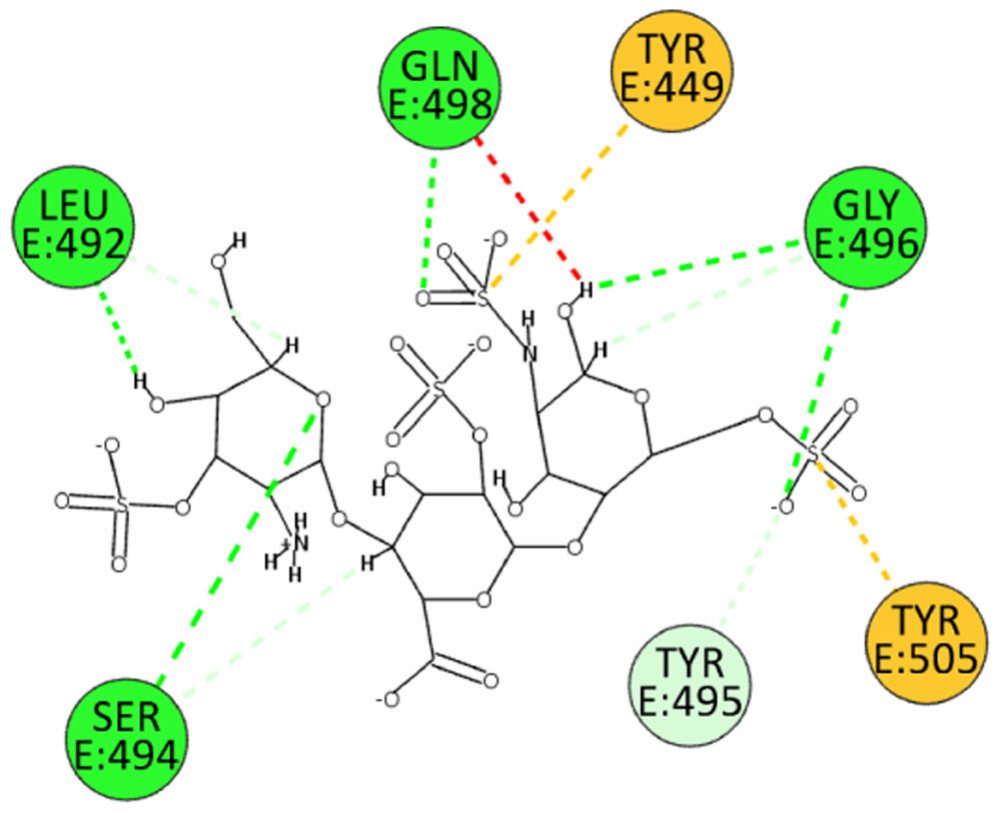
2D pose of HS at the interface between the crystal structures of SARS‐CoV‐2 spike bound to ACE2 (BA.2.86 variant).

We also performed docking experiments in the enzyme pocket of the BA.2.86 variant. The interactions between HS and the spike protein interface were similarly robust. HS forms hydrogen bonds with residues Leu492, Ser494, Gly496, and Gln498 through its sulfate and hydroxyl groups. Additionally, electrostatic interactions were observed with residues Tyr449 and Tyr505, and an unconventional hydrogen bond was established with residue Tyr495 (Figure 8). These interactions further support the strong binding affinity of HS and its potential efficacy against the BA.2.86 variant.

**Figure 9:**
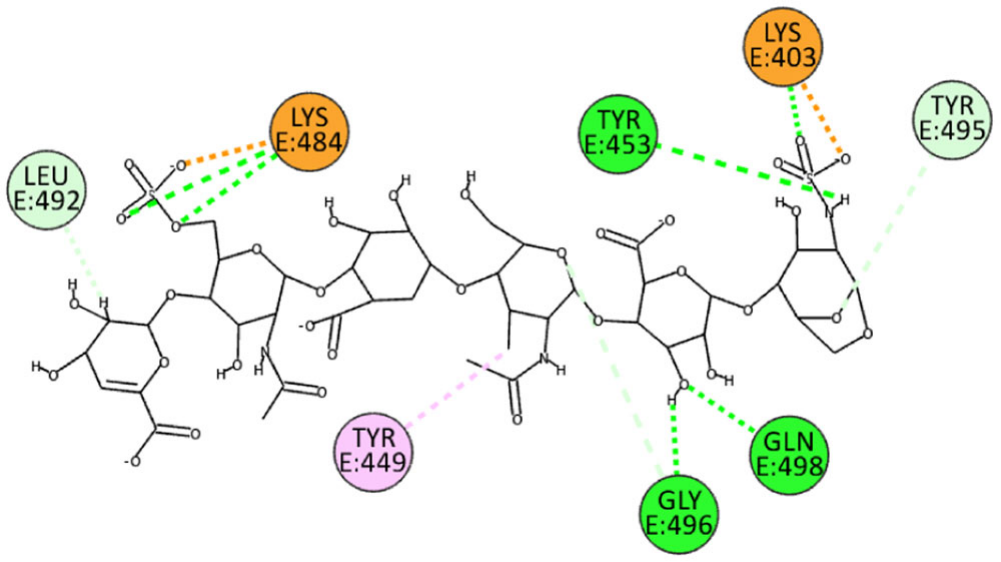
2D pose of EX at the interface between the crystal structures of SARS‐CoV‐2 spike bound to ACE2 (BA.2.86 variant).

EX exploits sulfate groups to bind at the interface through electrostatic bonds with residues Lys403 and Lys484. In contrast, exploiting both hydroxyl groups and nitrogen‐bonded protons, it establishes hydrogen bonds with residues Tyr453, Gly496, and Gln498. It also forms hydrogen and unconventional bonds with residues Leu492 and Tyr495 while establishing a *π*‐alkyl interaction with Tyr449 (Figure 9).

**Figure 10:**
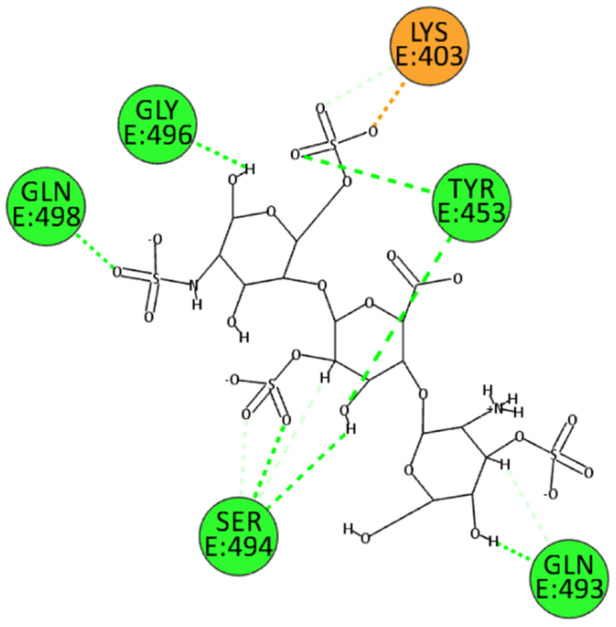
2D pose of HS at the interface between the crystal structures of SARS‐CoV‐2 spike bound to ACE2 (KP.2 variant).

**Figure 11:**
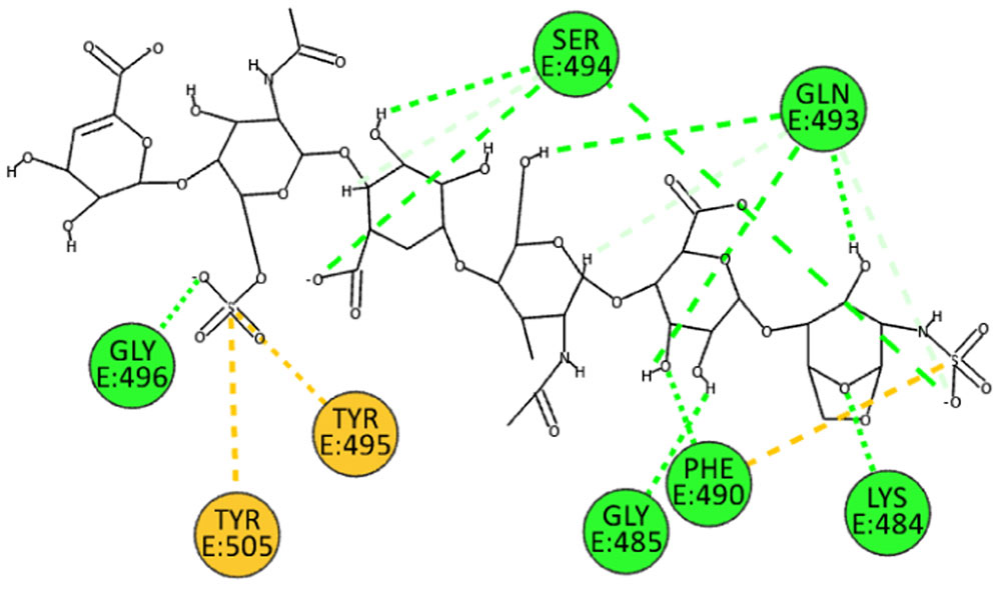
2D pose of EX at the interface between the crystal structures of SARS‐CoV‐2 spike bound to ACE2 (KP.2 variant).

**Figure 12:**
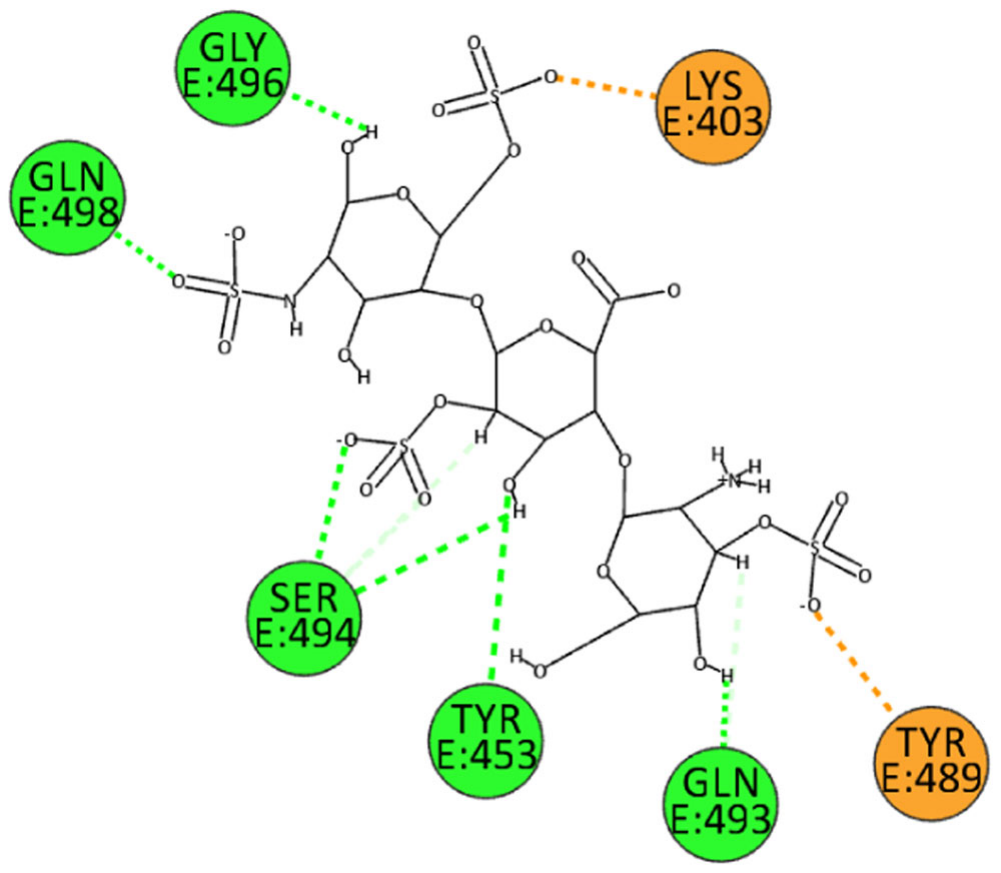
2D pose of HS at the interface between the crystal structures of SARS‐CoV‐2 spike bound to ACE2 (JN.1 variant).

**Figure 13:**
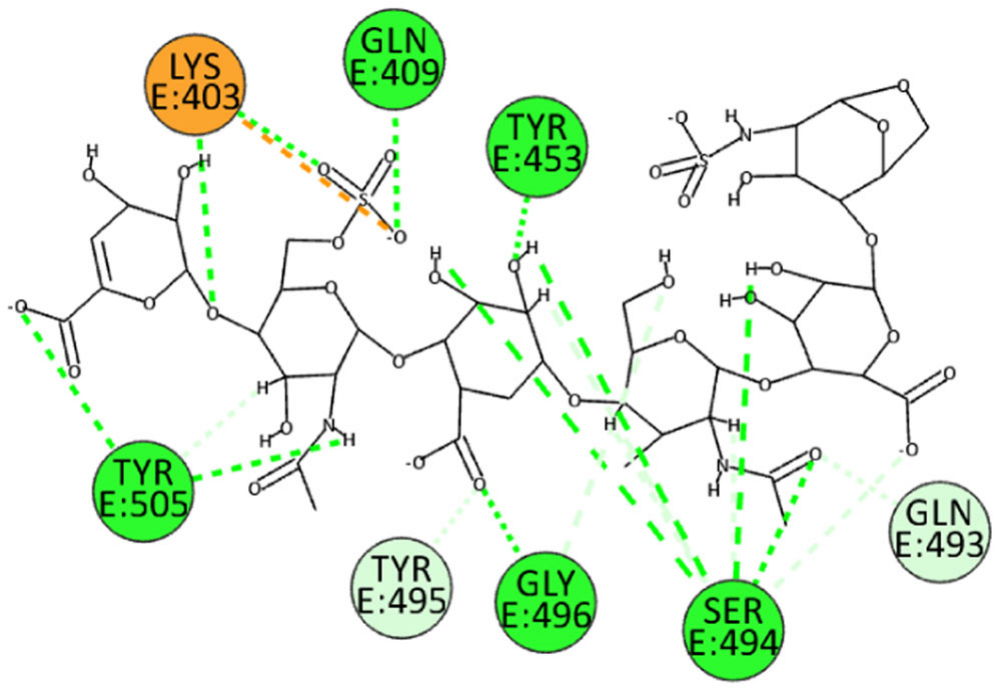
2D pose of EX at the interface between the crystal structures of SARS‐CoV‐2 spike bound to ACE2 (JN.1 variant).

The 2D poses of HS and EX at the interface of the two variants, KP2 and JN1, are shown in Figures 10, 11, 12, 13. From the 4 poses, it is easy to deduce that there is no significant difference in the interactions between HS and EX with the KP.2 and JN.1 variants compared with the wild type and the BA.286 variant.

The major interactions of HS and EX with the wild‐type interface and those of the BA.2.86, KP2, and JN1 variants are summarized in Table 1. In all eight experiments, the Gly496 residue constantly establishes hydrogen bonds with the ligands, suggesting it is a preferred anchoring site for both ligands.

**Table 1:** Summary of the main residuals between HS and EX with the two interfaces of SARS‐CoV‐2 spike, wild (6M0J), and variants (BA.286, KP.2, JN.1).

PDB ID: 6M0J		
	Attractive charge	Hydrogen bond
HS	Arg403, Tyr505	Tyr453, Ser494, Gly496, Gln498, Asn501
EX	Arg403, Phe456	Asn487, Tyr489, Gly496, Gln498

BA.2.86 variant		
	Attractive charge	Hydrogen bond
HS	Tyr449, Tyr505	Leu492, Ser494, Gly496, Gln498
EX	Lys403, Lys484	Tyr453, Gly496, Gln498

KP.2		
	Attractive charge	Hydrogen bond
HS	Lys403	Tyr453, Gln493, Ser494, Gly496, Gln498
EX	Tyr495, Tyr505	Lys484, Gly485, Phe490, Gln493, Ser494, Gly496

JN.1		
	Attractive charge	Hydrogen bond
HS	Lys403, Tyr489	Tyr453, Gln493, Ser494, Gly496, Gln498
EX	Lys403	Gln409, Tyr453, Ser494, Gly496, Tyr505

In summary, both HS and EX demonstrate consistent interactions with the Gly496 residue at the interfaces of all the studied isoforms from the wild type to the highly mutated BA.2.86, KP2, and JN1 variants, indicating their potential as robust antiviral agents against various forms of SARS‐CoV‐2.

## 3 CONCLUSIONS

The study investigated the antiviral efficacy of HS and EX sodium against SARS‐CoV‐2, focusing on their mechanisms of action and potential therapeutic applications. The findings highlight the significant role of these GAGs in inhibiting the virus's entry into host cells.

Despite the current plateau in SARS‐CoV‐2 infections, this virus has led to significant fatalities and economic losses.^[^ ^22^ ^]^ It remains crucial to thoroughly understand the variants and stay vigilant against new mutations, especially with the approaching winter, which may bring about a new wave of infections. Moreover, recent studies have presented conflicting results regarding the mechanisms of SARS‐CoV‐2 cell entry, raising scientific uncertainties and questions.^[^ ^23^ ^]^

Our *in*‐*vitro* experiments demonstrated that HS and EX exhibit substantial antiviral activity against SARS‐CoV‐2. The MTT assays revealed that HS is well tolerated by A549 cells even at high concentrations, whereas EX showed cytotoxicity at higher doses but was safe at lower concentrations. This suggests that both substances could be used at appropriate doses to inhibit viral entry without causing significant cytotoxic effects.

The concentrations of HS and EX used in this study were chosen based on their potential for therapeutic application, particularly in topical formulations for inhalation. This method of administration is relevant for respiratory infections, including those caused by coronaviruses. The selection of these concentrations aimed to balance efficacy and safety, ensuring that the compounds could exert antiviral effects without causing significant cytotoxicity. The concentrations tested (0.08–2.5 mg/mL) were sufficient to observe a reduction in viral cytopathic effect (CPE), particularly for HCoV‐229E, at the higher end of the range. This underscores the potential of HS and EX as antiviral agents against specific coronavirus strains, particularly those like HCoV‐229E.^[^ ^3^ ^]^ Using these compounds in topical formulations for inhalation could provide a targeted approach to mitigating respiratory viral infections. Administering these agents directly to the respiratory tract could enhance their antiviral efficacy while minimizing systemic exposure and potential side effects.^[^ ^24^ ^]^

HS plays a key role in the initial stages of SARS‐CoV‐2 infection by facilitating the virus's attachment to host cells. The study found that HS interacts electrostatically with the positively charged residues of the spike protein, enhancing the virus's concentration at the cell surface and increasing the probability of ACE2 receptor binding. This interaction is vital for viral entry, as evidenced by inhibiting viral transduction when cells were treated with HS. Molecular modeling confirmed strong binding interactions between HS and key residues on the spike protein, supporting its role as a potent inhibitor of viral entry. Indeed, Table 1 showed the calculated interactions in all the studied isoforms (6M0J wild type and BA.2.86 variant) despite the reported mutations for HS. Specifically, Gly496 and Gln498 were common residues for both wild and mutated forms in the case of EX. Therefore, despite the spike protein mutations, the calculated interactions and binding energies suggested that HS and EX should interact similarly with all the studied isoforms. However, in a previous study,^[^ ^3^ ^]^ HS and EX did not inhibit another *β*‐coronavirus, HCoV‐OC43, due to their relatively lower free binding energies compared with the original ligand, pyranosidonic acid (−6.40 kcal/mol). This suggests that the GAGs' mode of action is strongly ligand‐dependent and appears particularly effective in the spike‐ACE2 interaction, unlike other interactions not involving ACE2.

Our results provide compelling evidence of the antiviral potential of HS and EX against SARS‐CoV‐2. Both substances effectively inhibit viral absorption, with EX showing a slightly less pronounced effect than HS. While the reduction of infected cells with EX was significant, it was less dramatic than HS. Additionally, pretreating cells with HS or EX before virus exposure resulted in a robust antiviral effect, preventing the virus from entering the cells. HS exhibited complete inhibition of absorption, while EX showed a substantial 70% reduction, highlighting their potential as antiviral agents. Finally, cells exposed to the virus preincubated with HS or EX showed no signs of infection, indicating that these molecules may confer protection even before cellular exposure.

The updated literature supports our findings,^[^ ^7^, ^25^ ^]^ emphasizing the role of GAGs in viral entry and their potential as antiviral agents. The studies corroborate that HS and EX can interfere with the SARS‐CoV‐2 spike protein and ACE2 receptor interactions, aligning with our molecular modeling results and experimental data.

In conclusion, the study provides compelling evidence of the antiviral potential of HS and EX sodium against SARS‐CoV‐2. Both substances effectively inhibit viral entry through distinct mechanisms, demonstrating their utility as therapeutic agents. The findings support the strategic use of HS and EX in the prophylactic and therapeutic management of COVID‐19. Their ability to inhibit viral entry and reduce infectivity makes them promising candidates for further clinical development.

Ongoing research is essential to fully understand the efficacy and safety profiles of these GAGs, especially concerning emerging variants of SARS‐CoV‐2. Future studies should focus on detailed *in*‐*vivo* evaluations, dose optimization, and potential combination therapies to enhance their antiviral effects. The understanding gained from this study can be applied to other viral infections, given the role of GAGs in the infectivity of various viruses. This broader knowledge can lead to the development of novel antiviral strategies targeting viral‐host interactions.

In summary, HS and EX demonstrated significant promise as antiviral agents against SARS‐CoV‐2. Their mechanisms of action, supported by thorough experimental and modeling data, provided a strong foundation for further research and potential therapeutic applications in managing COVID‐19 and other viral infections.

## 4 EXPERIMENTAL

### 4.1 Chemicals, cellular lines, and viruses

HS and EX sodium were kindly supplied by Techdow Pharma S.r.l.; A549 CCL‐185™ (carcinoma lung epithelial cells) were purchased from American Type Culture Collection (ATCC). A549 cells were cultured in F12K medium supplemented with 2 mM l‐glutamine, 100 U/mL penicillin–streptomycin mixture, and 10% fetal bovine serum (FBS) at 37°C in a 5% CO_2_ humidified incubator.

Adherent subconfluent cell monolayers were prepared in growth medium (2% FBS) in 96‐well plates for cytotoxicity assays and viral inhibition tests. Pseudo SARS‐CoV‐2 Reporter was purchased from Montana Molecular and tested on A549 cells.

### 4.2 Cell viability assay by MTT

The evaluation of the cytotoxic effects of HS and EX on A549 cells was performed employing the MTT assay as previously reported.^[^ ^3^ ^]^ Briefly, cell viability was measured through the colorimetric reduction of MTT enzymatically catalyzed by the mitochondrial succinate dehydrogenase. The tetrazolium salts that enter the cells are transformed into violet‐colored formazan crystals. The level of formazan is used as an indirect index of cell density. The A549 cells were incubated with different concentrations of HS and EX for 24 and 48 h in 5% CO_2_ at 37°C. Then, 100 mL of the tetrazolium salt solution (0.5 mg/mL) was added to each well and incubated at 37°C in 5% CO_2_ for 3 h. After this incubation, 100 mL of DMSO was added, and the OD was measured at a wavelength of 570 nm with a spectrophotometer (BioTek Synergy HTX Reader). Three assays for each sample were performed, and the results were expressed as mean ± SD.

### 4.3 Transduction assay

A549 (1.0 × 10^4^/well) were seeded on 96‐well plates in complete F12K medium with 10% FBS and incubated overnight at 37°C in a humidified 5% CO_2_ atmosphere. Then, a red transduction mix of ACE2 (Santaka tagged), complete media, and sodium butyrate (Table 2) was prepared and used as described by the manufacturer (Montana Molecular, Fluorescent Biosensors for Live Cell Discovery). Briefly, 50 μL were added to each well of the plate. The plate was shaken 5–10 times to ensure uniform transduction and incubated at 37°C overnight. After 24–36 h of incubation, the transduction efficiency was evaluated by Leica DM IL LED (© 2023 Leica Microsystems). Finally, five different images were analyzed with LAS X Life Science Software. The experiment was performed thrice.

**Table 2:** ACE2 red transduction mix.

	Amount/well	Final concentration
ACE2 BacMam	5.0 μL	6.6 × 10^8^ VG/mL
Sodium butyrate	0.6 μL	2.0 mM
Complete media	44.4 μL	

Abbreviation: VG, vector genomes.

### 4.4 Adsorption inhibition assay

**Figure 14:**
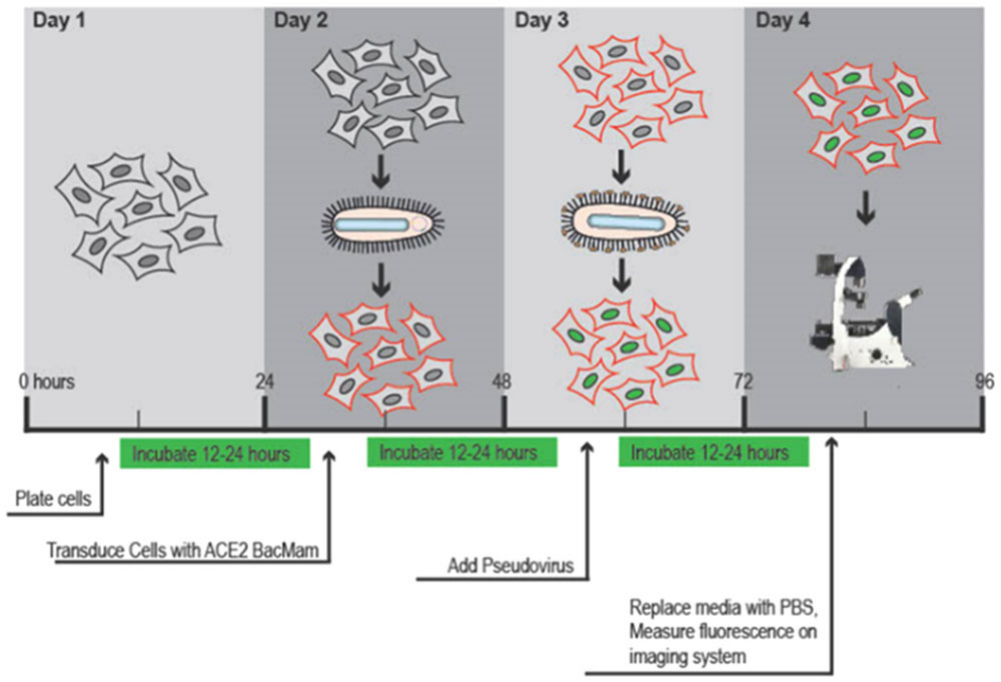
Transduction assay timeline.

A549‐ACE2 cells (1.0 × 10^4^/well) were seeded on 96‐well plates in complete F12K medium with 10% FBS and incubated overnight at 37°C in a humidified 5% CO_2_ atmosphere. The medium was then removed from the plate, and a fresh one containing HA and EX in concentrations equal to 5.0 and 1.25 mg/mL, respectively, was added. Cells were treated with the green transduction mix as described by the manufacturer (Montana Molecular, Fluorescent Biosensors for Live Cell Discovery), including pseudovirus (Neon‐Green tagged), fresh complete media, and sodium butyrate (Table 3). Figure 14 shows the timeline defined by the company's protocol.

**Table 3:** Pseudo‐SARS‐CoV‐2 green reporter transduction mix.

	Amount/well	Final concentration
SARS‐CoV‐2 pseudovirus	2.5 μL	3.3 × 10^8^ VG/mL
Sodium butyrate	0.6 mL	2.0 mM
Compound		5.0 mg/mL (HS) or 1.25 mg/mL (EX)
Complete media	adjust	

Abbreviation: VG, vector genomes.

**Figure 15:**
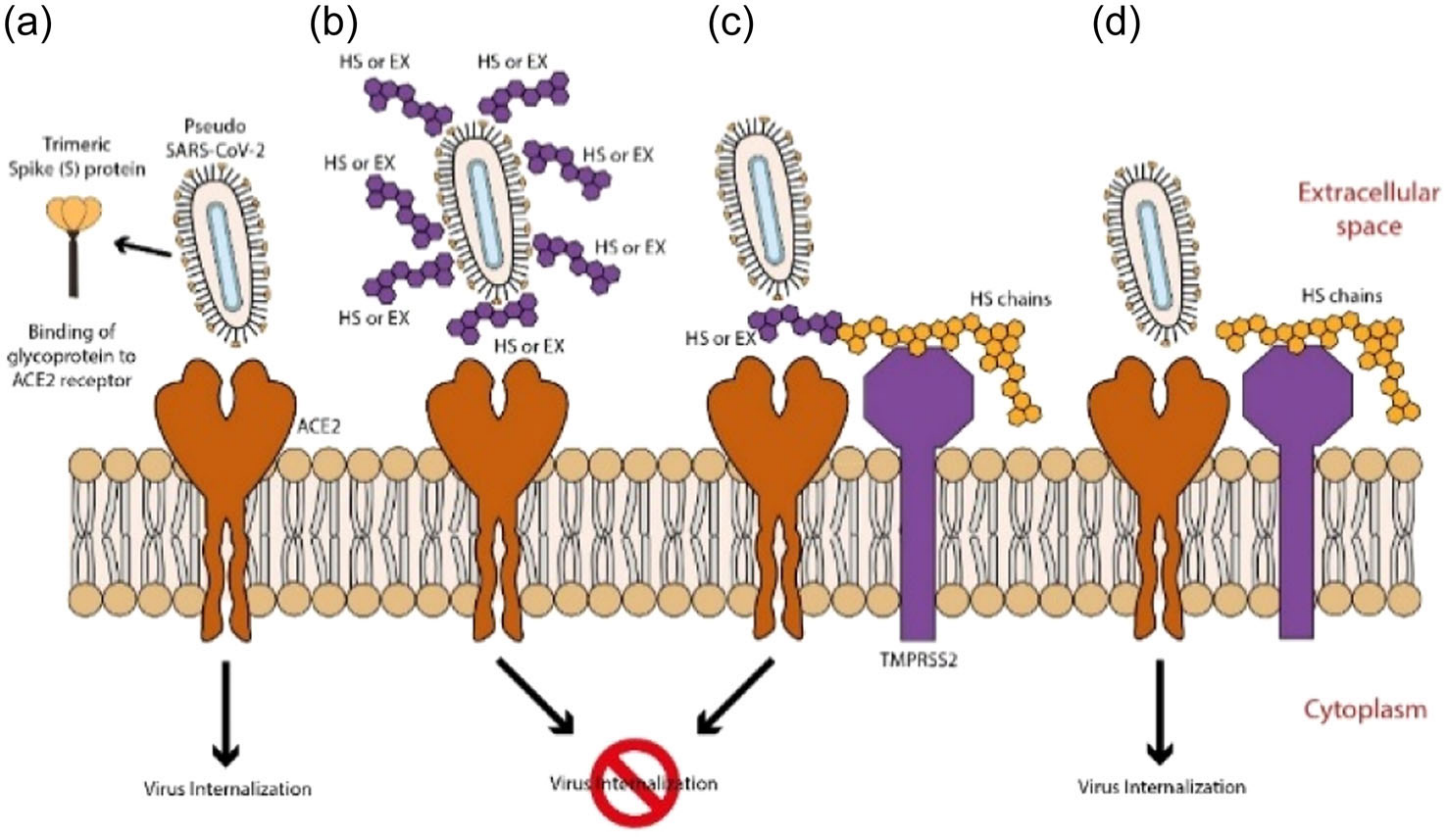
(a) ACE2 mediated viral entry; (b) HS or EX prevents viral entry by the creation of a molecular envelope that inhibits interaction between ACE2 receptor and Spike protein; (c) Heparan sulfate or enoxaparin linked to HS chains, constitutively present in cells membrane, hindered viral entry; (d) Viral entry mediated by ACE2 and HS or mediated by an alternative way mediated by HS only.

The experimental design for studying the antiviral activity of HS and EX involved dividing cells into three treatment groups based on the presumed mode of action of these molecules and the timing of dosing before viral adsorption (Figure 15). The treatment groups were as follows:

Group A (Co‐treatment): cells were treated with HS or EX simultaneously with viral adsorption. This setup allowed for examining the direct effect of these molecules on viral attachment and entry into the host cells.

Group B (Pretreatment with the substance): cells were pretreated with HS or EX before viral adsorption. This aimed to assess the potential preventive effect of these molecules on viral entry by modifying cell surface receptors or blocking viral attachment sites.

Group C (Pretreatment of the virus): the virus was pretreated with HS or EX for 2 h before infecting the cells. This setup investigated the impact of modifying viral surface components or inhibiting viral infectivity directly.

After the designated treatment period, the cell monolayer was washed twice with warm PBS in all treatment groups to remove unbound viral particles and excess treatment substances. Similarly, the same procedure was performed for the virus pretreated with the substances, ensuring that any unbound treatment substances were removed, allowing for accurate assessment of viral infectivity posttreatment. This step ensured that only virus particles successfully bound to the cells during the designated treatment period were included in subsequent analyses. Untreated A549 cells were used as the negative control to provide a baseline for viral infectivity without any treatment. This comparison allowed for assessing viral replication and infectivity levels between treated and untreated cells, aiding in evaluating the antiviral efficacy of HS and EX. Each experimental procedure is performed in quadruplicate to ensure statistical robustness and reproducibility of the results. This replication strategy minimizes experimental variability and enhances the reliability of the findings. After treating the cells and virus according to the experimental protocol, the plate was gently shaken 5–10 times to ensure uniform distribution of the treatment sub‐stances and viral particles across each well. Following shaking, the plate was placed in a standard cell culture incubator at 37°C with a humidified atmosphere containing 5% CO_2_. After 12–24 h of incubation, the efficiency of adsorption inhibition was assessed. This evaluation likely involved examining the extent of viral infection or replication within the treated cells compared with untreated control cells. The Leica DM IL LED microscope (© 2023 Leica Microsystems), equipped with appropriate fluorescence or phase‐contrast capabilities, was used for visualizing the cells and viral infection. Finally, five different images were analyzed with LAS X Life Science Software. All experiments were performed thrice.

### 4.5 Statistical analyses

All experiments were performed thrice, and data were summarized using the mean (±SD). Where applicable, data were analyzed by two‐way ANOVA with correction for multiple comparisons by Bonferroni. Graphs were generated using GraphPad® Prism ver. 8.4.2.0 (GraphPad Software) A *p* value ≤0.05 was considered significant.

### 4.6 Molecular modeling

Marvin Sketch was used to create the two molecules' 2D chemical structures, which were then all subjected to molecular mechanics energy minimization using the MMFF94 force field available in the same software (PubChem CIDs: 70678539 and 60196282).^[^ ^26^ ^]^ The PM3 Hamiltonian was then used to optimize the 3D geometry of all compounds,^[^ ^27^ ^]^ assuming a pH of 7.4.^[^ ^28^ ^]^ We tested different versions of the HS, as reported in the literature.^[^ ^29^ ^]^ Working with various variants produced no discernible differences. Since the side chains point outside the binding cavities and the main interactions are accomplished by the same portion of the molecule, they are irrelevant for interactions between proteins and heparan. Molecular docking experiments were achieved with AutoDock 4.2.6 and AutoGrid 4.2.6 provided in YASARA (v. 22.5.22, YASARA Biosciences GmbH)^[^ ^30^ ^]^ using the crystal structures of SARS‐CoV‐2 spike receptor‐binding domain bound with ACE2 (PDB ID: 6M0J) collected from the Protein Data Bank (PDB,http://www.rcsb.org/pdb, accessed on March 05, 2024) and an already validated protocol.^[^ ^31^ ^]^ The structure of the spike of BA.2.86 was created by manual single point mutations of the original spike protein as reported on the European Centre for Disease Prevention and Control website (https://www.ecdc.europa.eu/en/covid-19/variants-concern, accessed on March 06, 2024). The mutations were the following: I332V, D339H, R403K, V445H, G446S, N450D, L452W, N481K, 483del, E484K, F486P. The structure of the variants JN.1 (BA.2.86.1.1) and KP.2 (JN.1.11.1.2) were created by single point mutation accordingly with the mutation reported in the literature, particularly, the JN.1 variant, arising from BA.2.86 with the L455S substitution and KP.2 variant, a descendant of JN.1 bearing both R346T and F456L.^[^ ^21^ ^]^ The proteins have been optimized using YASARA software. The maps were made by AutoGrid (4.2.6) with a grid of 0.375 Å and an extension encompassing all atoms spanning 5 Å from the exterior of the structure of the ligand. Point charges were originally defined according to the AMBER03 force field and then damped to mimic the less polar Gasteiger charges used to optimize the AutoDock scoring function. All parameters were used at their default settings. In the docking tab, the macromolecule and ligand were selected, and LGA parameters were set as ga_cauchy_beta = 1.0, ga_mutation_rate = 0.02, ga_runs = 100, ga_crossover_mode = two points, ga_cauchy_alpha = 0.0, ga_pop_size = 150, ga_num_generations = 27,000, ga_crossover_rate = 0.8, ga_num_evals = 25,000,000, ga_elitism = 1, number of generations for picking worst individual = 10.

## CONFLICT OF INTEREST STATEMENT

The authors declare no conflict of interest.

## Data Availability

All data are in the manuscript.
